# The influence of commercial energy shots on response time and power output in recreational cyclists

**DOI:** 10.1186/s12970-014-0056-5

**Published:** 2014-12-10

**Authors:** John G Seifert, David A Connor

**Affiliations:** Movement Science Laboratory, Montana State University, Bozeman, MT 59717 USA

**Keywords:** Performance, Energy shots, Caffeine, Taurine, Carbohydrates

## Abstract

**Background:**

Caffeine based energy shot products accounted for $1.3 billion in sales in 2011. Caffeine has been shown to confer numerous benefits during exercise and is oftentimes combined with ingredients such as carbohydrates and taurine in the hope of further performance improvement. The purpose of this project was to compare auditory response time, power output, and physiological responses between the ingestion of a CHO, PRO, caffeine supplement (CPC), a caffeine-taurine-niacin based supplement (CTN), and a placebo (PL) in commercially formulated products that make claims as to improving performance.

**Methods:**

Fourteen subjects cycled an interval exercise of 70% VO_2_max for 13 minutes and 90% of VO_2_max for two minutes for a total of 120 minutes which was then followed by a six-minute power output (PO) task. Subjects ingested a total of 45 g CHO, 7.5 g PRO, and 375 mg caffeine for CPC while 512 mg caffeine and 1200 mg taurine were ingested for CTN throughout the exercise. The treatments were administered in a double blind, randomly assigned protocol. Response time was assessed by auditory response. Significance was set at p < 0.05.

**Results:**

Average PO was significantly greater for CPC: 309 ± 60 W than CTN: 290 ± 57 W and PL: 282 ± 63 W. Response time was significantly faster for the CPC: 0.219 ± .049 s than CTN: 0.232 ± .060 s and PL: 0.228 ± .047 s. HR was significantly greater for CTN: 143 ± 16 bpm than CPC: 139 ± 16 bpm. RPE was significantly lower for CPC: 13.0 ± 1.7 than CTN: 13.5 ± 1.2 and PL: 13.8 ± 1.9. Blood glucose was greater for CPC: 5.5 ± 0.8 mM/L than CTN: 4.9 ± 0.7 mM/L and PL: 4.6 ± 1.1 mM/L. No significant differences were observed for RER.

**Conclusions:**

The co-ingestion of CPC improved both cycling power output and auditory response time following 2 hours of moderate and high intensity interval cycling compared to CTN and PL. It is possible that the CPC treatment conferred not only a positive peripheral effect, but also a central effect. Even with a large caffeine dose, the combination of caffeine, taurine, niacin led to an inhibitory pattern which did not improve power output or response time performances over a PL.

## Background

Caffeine is a widely used supplement. Caffeine based energy shot products accounted for $1.3 billion in sales in 2011 [1]. In the athletic realm, Del Coso et al. reported that 74% of elite level athletes reported ingesting caffeine prior to competition [2]. Caffeine may confer numerous benefits such as improved endurance performance, strength performance, reaction time, fat oxidation, and a reduction in subjective perceived exertion [3-8]. As with caffeine research, the ingestion of carbohydrate during exercise has also been extensively studied. Endurance and high intensity performance and carbohydrate oxidation are generally improved while markers of central fatigue are mitigated with the ingestion of carbohydrates [9-13].

There has been interest in whether the combining of carbohydrates and caffeine would have a synergistic effect. The supplied carbohydrate serves as a potential substrate while caffeine serves as a neurological stimulant. Hulston and Jeukendrup reported that the co-ingestion of caffeine and carbohydrate led to a 4.6% improvement over carbohydrate-only solution in endurance performance [14]. Those authors reported that caffeine increases the oxidation of exogenous carbohydrate during exercise. Acker-Hewitt et al. reported a synergistic effect when carbohydrates and caffeine were combined compared to either of the components separately and a placebo during a short term, high intensity cycling exercise [15].

Most of the previous caffeine research has focused on supplying caffeine in a powder or capsule form. Additionally, caffeine has been typically dosed on a per kg body weight either before or during the exercise. It is unlikely, however, that athletes and recreational athletes dose their supplement ingestion based upon their body weight. Such dosing may not be truly representative of athletes’ habits during training or competition. Rather, products are typically ingested by a given serving size or volume of a given commercially available product.

It is quite rare, however, that one would find a caffeine-only product in the energy shot and drink market. Oftentimes, caffeine is combined with ingredients such as carbohydrates, taurine, and niacin in the hope of improving performance. Taurine is a non-essential amino acid which is found throughout the brain and skeletal muscle and serves as a neurotransmitter and neuromodulator [16,17]. In theory, combining caffeine and taurine, in the proper dose, should improve brain and muscle functions. Niacin is another common additive. It serves as a precursor to NAD and could have an influence in energy production.

Therefore, the purpose of this project was to compare auditory response time, power output, heart rate, and rating of perceived exertion between the ingestion of a carbohydrate + protein + caffeine supplement (CPC), a caffeine-taurine-niacin based supplement (CTN), and a non-caloric placebo in commercially formulated products that make claims as to improving performance.

## Methods

Following approval from the Montana State University IRB, 14 subjects provided informed consent to participate in this project. Subjects’ average age was 30.1 ± 3.9 y while average weight 74.9 ± 9.5 kg. Four females (mean weight and VO_2_max were 66.7 ± 6.2 kg and 42.8 + 2.3 mL/kg/min) and 10 males (mean weight and VO_2_max were 80.8 ± 6.4 kg and 51.6 ± 7.0 mL/kg/min) participated. Oxygen uptake was assessed using a three-minute ramped protocol to volitional exhaustion with the highest attained VO_2_ used to calculate experimental workloads. All subjects were recreational cyclist who exercised regularly, but did not compete in races.

The two experimental treatments were a commercially available sports CPC shot containing carbohydrate, protein, and caffeine (Body Glove Surge®, 18 grams CHO, 3 grams PRO, and 150 mg caffeine per serving; PacificHealth Laboratories, Inc., Matawan, NJ), a commercially available caffeine-based shot (5 Hour Energy®; ~205 mg caffeine, ~480 mg taurine, 30 mg niacin per 57 mL serving; Living Essentials, LLC, Farm Hills, MI), and a non-caloric liquid placebo (PL) served as the control. Total caffeine ingested during the exercise was 375 mg for CPC and 512 mg for CTN. Subjects ingested a half of a serving (28 mL) at 30 minutes of exercise and then one serving (57 mL) of the given treatment after 60 minutes of exercise and then another serving (57 mL) after 90 minutes of exercise. The goal of this administration was an attempt to have maximal caffeine levels for the performance tests at the end of the two hour exercise.

Along with the given treatment, subjects also ingested 75 mL plain water. Treatments were administered in a double blind, randomly assigned fashion in a crossover, counterbalanced protocol. Seven to 10 days elapsed between experimental days. The PL was flavored similar to that of CPC and CTN. To further blind the subjects to the treatments, multiple flavors of each treatment were used.

**Figure 1:**
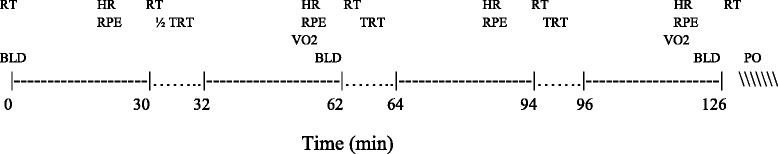
**Schematic of exercise protocol.** Mean ± SD; RT: auditory response time, HR: heart rate, RPE: rating of perceived exertion, ½ TRT: half dose of treatment, VO2: oxygen consumption collection, BLD: blood collection, TRT: full dose treatment, PO: six minute power output test.

Subjects cycled for 120 minutes which was then followed by a six-minute time trial performance task (see Figure 1 for a schematic of the protocol). The 120 min segment was divided into eight - 15 minute intervals. Each interval required the cyclists to pedal at a workload corresponding to 70% of their maximal oxygen uptake for 13 minutes followed by cycling for two minutes at a workload corresponding to 90% of their maximal oxygen uptake. Heart rate (HR) and rating of perceived exertion (RPE) were collected at the 28 minute mark of each 30 minute phase. Rating of perceived exertion (RPE) was collected on the 6–20 Borg scale. Each 30 min phase was separated by a two minute break. Following the 120 minute reaction test, subjects completed a time trial test where they attempted to produce and maintain as great of power output as possible during the six minute test. A Monark 868 ergometer and SMI Power program were used to assess power output. Cycling resistance was set at 5% of BW.

During each 30 min break, an auditory response time test was completed by the subjects. Subjects dismounted the ergometer and sat in a nearby chair to complete the test. Subjects wore headphones that were connected to a computer. A computer based program was used to assess auditory response time (BioPac Systems, Coleta, CA). The response time test is a simple auditory response time test where subjects pushed a switch upon hearing 10 randomly spaced beeps. Times were analyzed by computing the average of the 10 beeps that were collected through the program. All subjects practiced and were experienced with the timing apparatus for this test.

Blood glucose was monitored by a fingerstick sample taken pre-exercise, 60, and 120 minutes (Bayer Contour, Whippany, NJ). Blood samples were measured in duplicate with the mean used for analysis. Expired air was collected for five minutes at 55 minutes and 110 minutes with data from the final three minutes of each interval averaged and analyzed for respiratory exchange ratio (RER; ParvoMedics, Salt Lake City, UT).

Subjects entered the lab four hours post prandial. Laboratory temperature was 21°C with 28% RH. All subjects ingested caffeine in their diets at some point during their normal day. The lowest intake was about 90 mg/day while the highest was about 270 mg/day. Subjects were instructed to consume similar diets and minimize exercise for 24 hour prior to each of their trials. Caffeine ingestion was not allowed on the day of testing. All subjects exercised either in the late morning or early afternoon to minimize diurnal variation.

Data were analyzed using a repeated measures ANOVA. Upon a significant interaction, Tukey’s post hoc test was used to differentiate means at a given time point. Significance level was set at p < 0.05. All data are listed as mean (±SD).

## Results

Data from one subject were not included in the analyses as he entered his final trial extremely dehydrated. He did not complete the final trial. Therefore, data was analyzed using 13 subjects.

### Power output

**Figure 2:**
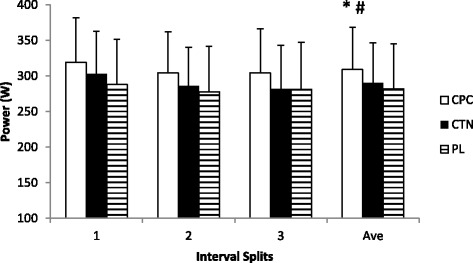
**Power output.** Mean ± SD; *: main effect of treatment, CPC is significantly faster than CTN and PL.

Subjects ingesting CPC completed the 6 min time trial with a statistically greater average power output than when ingesting CTN and PL (Figure 2). Average power outputs during the time trial were 309.1 ± 60 W for the CPC trial, 290.2 ± 57 W for CTN trial, and 282.4 ± 63.1 W for PL trial. These differences amounted to 6.5% between CPC and CTN while the difference between CPC and PL was 8.8%. No statistical difference was observed between CTN and PL (p = .08). No significant differences were observed for the main effect of time or for the interaction of treatment by time.

### Response time

**Figure 3:**
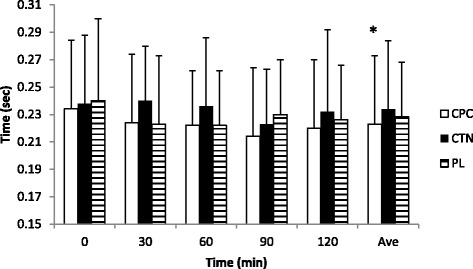
**Auditory response time.** Mean ± SD; *: main effect of treatment, CPC is significantly faster than CTN and PL.

Average auditory response time for the CPC trial was significantly faster than that of CTN and PL trials (Figure 3). Average response times were 0.219 ± 0.049 s for the CPC trial, 0.232 ± 0.060 s for CTN, and 0.228 ± 0.047 s for the PL trial. As with power output, no statistical difference was observed between CTN and PL (p = 0.06). The differences amount to 5.7% between CPC and CTN, 2.2% between CPC and PL, and 2.6% between CTN and PL. No significant differences were observed for the main effect of time or for the interaction of treatment by time.

### Heart rate

**Figure 4:**
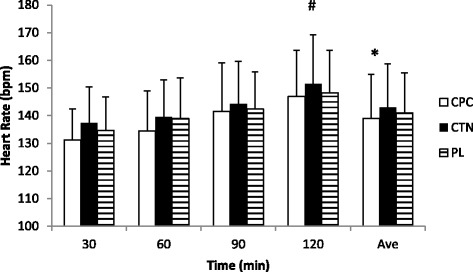
**Heart rate during experimental ride.** Mean ± SD; #: main effect of time, group average is significantly greater than 30 min group average. *: main effect of treatment, average HR for CPC is significantly lower than CTN.

Over the course of the 120 min exercise, the ingestion of the CPC led to a significantly lower average HR than CTN (Figure 4). Average HR for the CPC trial was 138.5 ± 16.0 bpm, 143.1 ± 15.6 bpm for CTN, and 141.0 ± 14.5 bpm for PL. No difference was observed between CPC and PL (p = 0.08) or for CTN vs. PL (p = 0.09). No interaction of treatment by time was observed. There was however, a main effect of time was observed where the average HR value at 120 min was significantly greater that at 30 min.

### RPE

Average RPE for CPC, CTN, and PL were 13.0 ± 1.7, 13.5 ± 1.2, and 13.8 ± 1.9, respectively. Rating of perceived exertion for the CPC was significantly lower than CTN and PL. There was no difference between CTN and PL (p = 0.1).

### Blood glucose and RER

Blood glucose (Table 1) at 120 min was significantly greater for CPC (5.5 ± 0.8 mMol/L) than CTN (4.9 ± 0.7 mMol/L) and PL (4.6 ± 1.1 mMol/L). No difference was observed between CTN vs. PL at either time point for blood glucose. No differences were observed between treatments, or time, for RER during the exercise (Table 1). Average RER for CPC was 0.87 ± 0.04), 0.87 ± 0.04 for CTN, and 0.86 ± 0.04 for PL.

**Table 1:** **Blood glucose and respiratory exchange ratio values during 120 min of cycling**

	**0 minutes**		**60 minutes**		**120 minutes**	
	**BG**	**RER**	**BG**	**RER**	**BG**	**RER**
CPC	4.6 (0.5)	-	5.0 (0.7)	0.87 (0.04)	5.5 (0.8)*	0.87 (.04)
CTN	5.0 (0.6)	-	4.8 (0.4)	0.88 (0.03)	4.9 (0.7)	0.86 (.04)
PL	4.6 (0.6)	-	4.7 (0.7)	0.88 (0.04)	4.6 (0.5)	0.85 (.04)

CPC: carbohydrate, protein, and caffeine supplement, CTN: Caffeine, taurine, and niacin supplement; PL: Placebo; BG: blood glucose (mMol/L), RER: respiratory exchange ratio; Mean (±SD); *: significantly different from CTN and PL.

## Discussion

The primary purpose of this study was to investigate the effects of commercially available products, containing caffeine, on response time and cycling performance. The major finding of the present study is that a low dose carbohydrate, protein, and caffeine supplement (22.5 g/hr CHO, 3.5 g/hr PRO and 188 mg caffeine/hr) resulted in improved power output and response time over that of CTN treatment (256 mg caffeine/hr) even though the CPC contains 27% less caffeine than the CTN treatment. In the present study, 10 of 13 subjects had a greater power output during the time trial when ingesting CPC compared to CTN, while 12 out of the 13 subjects had a significantly faster average response time at 120 min during the CPC trial compared to CTN. In contrast to previously published reports, even with the high dose of caffeine, the CTN treatment did not confer statistically significant power output or response time performance advantages over PL [5,18,19], although there were strong trends for CTN to result in improved performances (p = 0.08 and p = 0.06, respectively). However, the current results do support other reports that have shown no significant difference between caffeine and placebo [5,20,21].

Results of the present study support previous findings that there is a synergistic effect of the co-ingestion of CHO and caffeine on performance [14,15,22]. Power output was improved in the present study with CPC ingestion by 6.5% over CTN and 8.8% over the PL. Hulston and Jeukendrup reported that the co-ingestion of caffeine (5.3 mg/kg BW) with CHO during exercise enhanced the 45 min time trial performance by 4.6% compared with CHO only and 9.0% compared with a water placebo [14]. Those authors noted that caffeine increased exogenous CHO oxidation and glucose kinetics during steady state exercise. The results of the present study are rather surprising given the fact that the CTN treatment provided 256 mg/hr of caffeine while the CPC provided 188 mg/hr of caffeine. However, as Hulston and Jeukendrup [14] and Yeo et al. [22] reported, caffeine increases the oxidation of exogenous carbohydrate which would point to the fact that more of the CHO supplied by the CPC was oxidized for fuel, perhaps changing the endogenous substrate input.

The improvement in performance with the CPC in the present study cannot be explained by changes in RER, although blood glucose concentration was greater with the CPC than the CTN and PL trials. The RER data supports those findings reported by Graham and Spriet. [23] and Graham et al. [24] who reported that 9 mg/kg and 6 mg/kg caffeine ingestion did not alter RER during endurance exercise compared to a placebo. Yeo et al. reported that co-ingestion of 5 mg/kg BW caffeine with 48 g/h of CHO increased the rate of the exogenous carbohydrate oxidation during 120 min of cycling at 64% VO_2_max [22]. Additionally, Van Nieuwenhoven et al. reported that caffeine augments carbohydrate kinetics [25]. It is plausible that CHO uptake from the gut and movement into the active muscle may be enhanced when caffeine and CHO are combined. However, previous metabolic findings do not support those of the present study where CTN ingestion did not lead to differences in substrate metabolism between treatments as assessed by RER and blood glucose levels. Thus, there must be an confounding interaction of ingredients in CTN.

While it was expected that the CPC treatment would most likely improve performance, due to the CHO content, it was surprising that the CTN treatment did not improve power output or response time performance over the PL, in contrast to numerous other studies [3-8,26,27]. Although the present study did not attempt to establish the mechanism of performance changes, there are a number of possibilities as to why performance was not improved with CTN. The 6.2 mg/kg and 7.5 mg/kg caffeine doses in the present study are on the high end of dosing that has been reported to induce positive results [4,26,27]. There is the possibility that the combination of taurine and niacin found in CTN minimized the influence of caffeine. Jia et al. [28] demonstrated that taurine reduced the excitability of thalamocortical relay neurons and activated the extrasynaptic GABA_A_ receptors. GABA receptors are well known as inhibitory receptors. These authors reported that taurine actually exhibits a sedative effect on the brain. Barthel et al. noted that taurine neutralized the positive effects of caffeine in the premovement brain potentials in both the frontal and parietal regions [29]. Additionally, Lin et al. reported that a high ratio of taurine to caffeine induced lethargy and sleepiness [30]. The CTN product used in the present study contains 476 mg of taurine and 207 mg caffeine per serving which would be considered a high ratio. It was also expected that RER would favor fat oxidation with CTN. However, this was not the case. It is possible that niacin in CTN exhibited a negative influence on physiological and performance by altering the FFA response during exercise [31,32]. Thus, it is plausible that any positive effects of caffeine ingestion on response time and power output were neutralized by the addition of taurine and niacin to the CTN product.

The present study opted to use an auditory system to assess central fatigue. This would minimize the influence of gross motor movement patterns on this type of performance. Souissi et al. [33] fed subjects 5 mg of caffeine/kg BW and found that visual reaction time increased as well as peak and mean power with caffeine ingestion compared to the placebo trial. That study was performed with subjects completing a 30 sec Wingate test in the early morning hours without consuming breakfast. Subjects in the Souissi et al. [33] study also improved reaction time with caffeine ingestion by about 11% over the placebo trial. In contrast, subjects in the present study were four hours post prandial while response time was improved for the CPC treatment by nearly 6% over the CTN treatment. Ingredients of the supplements, exercise intensities (Wingate vs. 2 hr interval work), pre-exercise feeding minimizes the effect of caffeine on muscle energetics, and the type of response test used (visual vs. auditory) all may explain the differences in these two studies’ results.

## Conclusions

In conclusion, the present study attempted to mimic real life conditions by providing commercially available energy shots in doses that are used in ‘real world’ settings, by allowing subjects to be fed four hours before their experimental rides, and by assessing performance based on measuring peripheral, or muscular, and central fatigue. Although other rigorous assessments of central fatigue were not used, the use of auditory response time added practicality to the study. Study results demonstrate that, following two hours of moderate and high intensity interval cycling, ingestion of carbohydrate, protein and caffeine containing shot produced positive peripheral and central effects by significantly improving cycling power output and auditory response time compared to a caffeine-taurine-niacin shot and a non-caloric placebo. While response time is a novel measure of central fatigue, it may be considered a limitation of this study when compared to other tests. The expected responses on power output and response time were not observed when caffeine was combined with taurine and niacin. Although not statistically significant different from PL, there was a strong trend for CTN to result in improved performance. This poses the practical significance implications of this supplement. Additional research of commercial supplements is needed to not only further elucidate performance characteristics, but also on the mechanisms of possible antagonistic interactions. However, the results of these studies indicate that the combination of carbohydrate, protein, and caffeine improves exercise performance and would be of benefit for individuals participating in moderate to intense exercise. The caffeine-taurine-niacin combination may be problematic since the results of the present study indicate no performance improvement, either peripherally or centrally, when compared to a non-caloric placebo.
